# The Slt2-Swi6 signaling pathway plays a pivotal role in regulating cell wall biosynthesis, secondary metabolite production, and the pathogenicity of pear fungal *A. alternata*

**DOI:** 10.1080/21505594.2026.2715208

**Published:** 2026-08-05

**Authors:** Rong Li, Yiyang Liu, Li Li, Yanxia Zhang, Xiwang Wang, Xu Ji, Miao Zhang, Yuanyuan Zong, Yang Bi, Yongcai Li

**Affiliations:** College of Food Science and Engineering, Gansu Agricultural University, Lanzhou, PR China

**Keywords:** CWI, *Alternaria alternata*, transcription factor, mycotoxin, pathogenicity

## Abstract

The cell wall integrity (CWI) MAPK signal pathway is crucial for the assembly of fungal cell walls and their associated virulence, as it activates the expression of the downstream transcription factors Mbp1, Swi6, and RlmA. However, the transcription factors directly regulated by this pathway in Alternaria alternata have yet to be identified. In this study, we delineated the functional roles of two transcription factors, AaSwi6 and AaMbp1, through targeted gene deletion. Disruption of either gene resulted in impaired hyphal extension, reduced mycelial accumulation, decreased conidiation, altered differentiation of infection structures, and impaired melanin synthesis. Furthermore, when compared to the WT, the levels of alternariol (AOH), alternariol monomethyl ether (AME), and tenuazonic acid (TeA) toxins in the two ΔAaSwi6 and ΔAaMbp1 mutants decreased by 63.98% and 86.32%, 36.71% and 82.85 %, and 33.28% and 33%, respectively. These defects were correlated with diminished virulence on tomato and pear fruit. Compositional analysis of the cell wall composition indicated that the deletion of AaSwi6 resulted in decreased levels of chitin, glucan, and mannan, whereas the deletion of AaMbp1 led to reduced levels of chitin and mannan. Yeast two-hybrid experiments further demonstrated that the MAPK kinase AaSlt2 physically interacted with downstream partners AaSwi6 and AaRlmA, and that AaSwi6 also interacted with AaMbp1 and AaRlmA. In conclusion, our study identifies a physical interaction between AaSlt2 and Swi6/RlmA, suggesting that these components are critical for cell wall synthesis. The finding advances our understanding the pathogenic mechanisms of A. alternata and proposes potential strategies for controlling postharvest diseases.

## Introduction

*Alternaria alternata*, a widely distributed phytopathogenic fungus, infects over 100 plant species, leading to significant deterioration of agricultural produce and severe economic impacts. Throughout its infection cycle, the fungus releases mycotoxins that pose serious threats to human health [1–3]. These Alternaria toxins (ATs), which are indispensable for fungal colonization and pathogenicity, are classified into two major categories: host-specific toxins (HSTs) and non-host-specific toxins (nHSTs). Generally, nHSTs exhibit a broader pathogenic activity, with the most prevalent being alternariol monomethyl ether (AME), alternariol (AOH), tenuazonic acid (TeA), and tentoxin (TEN) [4,5]. Moreover, during host invasion, fungal pathogens secrete a variety of cell wall degrading enzymes (CWDEs) that disrupt plant structural integrity, thereby facilitating colonization and enhancing virulence [6]. *A. alternata* is particularly noted for producing diverse pectin-degrading enzymes, along with cellulases and cutinase, which significantly contribute to its pathogenic success [7]. Therefore, a comprehensive understanding of the developmental mechanisms and pathogenic processes of the phytopathogenic fungus *A. alternata* can provide a theoretical foundation for disease prevention and control.

Preserving the integrity of the cell wall is paramount for fungi to withstand environmental stresses and effectively invade host tissues [8]. Composed predominantly of chitin, glucan, mannan, melanin, GPI-anchored hydrophobic proteins, and various extracellular materials, the precise architecture of the cell wall varies among plant-pathogenic fungal species [9]. A central system overseeing the biosynthesis and maintenance of this protective barrier is the cell wall integrity (CWI) pathway. This signaling cascade detects perturbations in cell wall homeostasis caused by environmental stimuli and is indispensable for fungal virulence [10]. Highly conserved in fungi, the CWI pathway was originally characterized in *Saccharomyces cerevisiae*. The signaling cascade initiates with membrane-localized sensors that activate the small GTPase Rho1, triggering a phosphorylation cascade either through Pkc1 or by the direct activation of Bck1, culminating in Slt2 MAPK phosphorylation. This process involves the translocation of the MAPK into the nucleus to modulate transcription factors, thereby regulating the expression of genes involved in cell wall remodeling and virulence [11,12]. The CWI pathway has been identified in several phytopathogenic fungi, including *Fusarium oxysporum*, *Magnaporthe oryzae*, *Aspergillus fumigatus*, *Botrytis cinerea*, and *A. alternata*, indicating its conserved role in fungal pathogenesis. Functional studies in these organisms have linked the CWI pathway to a broad spectrum of biological processes, including hyphal growth, conidiation, cell wall stress adaptation, phosphorylation-dependent regulation, mycotoxin biosynthesis, and pathogenicity [10,13–16].

Activation of the CWI pathway triggers a coordinated transcriptional reprogramming in fungal cells, primarily mediated by the transcription factor Rlm1, along with Swi4 and Swi6 [17]. Rlm1 belongs to the MADS-box transcription factor superfamily, while two APSES-type transcription factors, Mbp1 (the Swi4 homolog) and Swi6, form the MBF complex [18,19]. In *Cytospora chrysosperma*, CcRlm1 significantly influences hyphal growth, conidiation, cell wall biosynthesis, and virulence [20]. Similarly, *Arthrobotrys oligospora* requires *AoSwi6* for normal growth, stress adaptation, cell wall stability, and pathogenicity [21]. The Mbp1-Swi6 complex in *F. verticillioides* contributes to pathogenicity, vegetative growth, and stress tolerance [19]. As demonstrated in our previous study, Δ*AaRlmA* affects biological functions such as growth and development, cell wall synthesis, secondary metabolite production, and pathogenicity in *A. alternata* [18]. Nevertheless, the specific biological roles of the two APSES type transcription factors Mbp1 and Swi6 in *A. alternata* have not yet been clarified, and it is still unknown whether Swi6 and Mbp1 act as the main downstream effectors of the CWI signaling pathway Slt2, coordinating cell wall remodeling and virulence gene expression.

This study constructed Δ*AaSwi6* and Δ*AaMbp1* mutants, revealing that these two transcription factors are involved in regulating the growth of *A. alternata*, as well as the differentiation of infection structure, stress responses, and pathogenicity. Results from Yeast two-hybrid (Y2H) analysis indicated that MAPK AaSlt2, the component of the CWI signaling pathway mediates cell wall synthesis through its interaction with AaSwi6 and AaRlmA rather than with AaMbp1. Furthermore, AaSwi6 interacted with both AaRlmA and AaMbp1, which facilitates further specific functional activities. These findings elucidate the biological roles of *AaSwi6* and *AaMbp1*, revealing how the CWI pathway may regulate downstream transcription factors. Furthermore they provide new mechanistic insights into the regulatory mechanisms governing growth, development, and pathogenicity in pathogenic fungi.

## Materials and methods

### Fungal strains and culture conditions

The wild-type (WT) strain of *A. alternata* was isolated from the decayed “Zaosu” pear fruit (*Pyrus bretchneideri*) and characterized by sequencing the 16S rRNA. The fungal strains were cultured on potato dextrose agar (PDA) medium at 28°C in the dark.

### AaMbp1 and AaSwi6 orthologues in *A. alternata*

Based on the characterized protein sequences of *F. verticillioides* Swi6 and Mbp1 [19], we performed a BLAST search in the NCBI database to obtain the protein sequences of these two transcription factors in *A. alternata*, constructe phylogenetic trees of these two transcription factors from different fungi using Clustal and MEGA 7.0 software, and analyzed the phylogenetic relationships of different fungi, and used Conserved Domain Search online tool (https://www.ncbi.nlm.nih.gov/cdd) to predict conserved domains.

### Construction of gene deletion strains

To investigate the functions of *AaSwi6* and *AaMbp1* in *A.alternata*, we designed specific primers targeting their coding sequences using Premier 5.0. Target gene deletion vectors were constructed following the protocol described by Li et al. [18]. The *A. alternata* protoplast transformation was performed using polyethylene glycol (PEG)-mediated methodology, yielding putative transformants. These were initially screened by PCR amplification and subsequently validated through RT-qPCR analysis of transcript levels (Fig. S1), the primer sequences are detailed in Table S1.

### Phenotype determination

#### Vegetative growth, biomass accumulation, and sporulation

Mycelial plugs from each strains were inoculated onto three types of media: complete medium (CM), potato dextrose agar (PDA), and minimal medium (MM). Cultures were incubated at 28°C in darkness for 5 d, after which colony morphology was documented photographically and diameters measured using the cross method. To determine biomass, inoculate each strains on PDA medium covered with cellophane of the same weight at 28°C for 6 d, and then weigh each strain. For sporulation assays, 2 µL of spore suspensions from each strain was spotted onto PDA plates and incubated at 28°C for 6 d. Harvesting was performed by adding 5 mL sterile aqueous solution containing 0.01% Tween 80, followed by spore quantification using a hemocytometer [22].

### Infection ability assay

Spore germination and appressorium-like formation rates were determined following the protocol established by Zhang et al. [23]. Briefly, 20 µL spore suspension was placed on GelBond hydrophobic membranes (Youningwei Biotechnology Co. Ltd., Shanghai, China, 80,112,936) mounted on glass slides. Samples were maintained at 28°C with 95 % relative humidity, with spore germination rates quantified after 3 h and appressorium-like formation rates assessed following 6 h of incubation. The infection‑related morphogenesis in the *A. alternata* is shown in Fig. S2.

### Stress response assays

To assess stress tolerance, spore suspensions were inoculated onto PDA plates amended with various stress compounds: 1 M NaCl (Sinopharm Chemical Reagent Co., Ltd., Shanghai, China, 10,019,318), 1 M sorbitol (Sinopharm Chemical Reagent Co., Ltd., Shanghai, China, 63,011,037), 200 μM Congo red (Sinopharm Chemical Reagent Co., Ltd., Shanghai, China, 71,011,160), 0.02 % Sodium Dodecyl Sulfate (SDS, Sinopharm Chemical Reagent Co., Ltd., Shanghai, China, 30,166,428), 50 μg/mL calcofluor white (CFW, Sigma-Aldrich, USA, 910,090), 5 mM H_2_O_2_ (Sinopharm Chemical Reagent Co., Ltd., Shanghai, China, 10,011,218), or 60 μM menadione (MAD, Sinopharm Chemical Reagent Co., Ltd., Shanghai, China, XW015827502). Following 5 d of incubation at 28°C, colony diameters were measured using the cross method and documented photographically.

### Determination of cell wall component contents

Cell wall component analysis in *A. alternata* was conducted following Li et al. [24]. Briefly, 0.1 g mycelia from 6 d PDA cultures were homogenized in phosphate-buffered saline (PBS, Solarbio Science & Technology Co., Ltd., Beijing, China, P1020), centrifuged at 12,000 rpm for 10 min to obtain the supernatant. The contents were determined by fungus Chitin ELISA Kit instruction, fungus Glucan ELISA Kit instruction, and fungus Mannan ELISA Kit (SinoBest Biotechnology Co., Ltd., Shanghai, China, YX-23023F, YX-23210F, YX-23216F). All assays were performed in triplicate. For chitin visualization, spores were stained with CFW for 15 min and examined under fluorescence microscopy (Olympus, excitation 365 nm).

### Determination of DHN melanin content

For extracellular melanin determination, fungal colonies from 6 agar plates cultured for 6 d at 28°C in darkness were transferred to 100 mL PDB and incubated at 28°C with shaking (150 rpm) for 6 d. The culture filtrate was adjusted to pH 2.0 using 7 M HCl (Sinopharm Chemical Reagent Co., Ltd., Shanghai, China, 100,110,186). Following centrifugation at 10,000 rpm for 15 min, the supernatant was completely discarded to obtain the crude melanin extract. Subsequently, 7 M HCl was added to the precipitate, and hydrolysis was performed in a boiling water bath for 2 h to further purify the melanin. Centrifugation was then repeated to remove the supernatant, after which the residue was dissolved in 1 M NaOH (Sinopharm Chemical Reagent Co., Ltd., Shanghai, China, 10,019,764) and repeated acid-base purification was conducted. Finally, the solution was diluted to a final volume of 10 mL with 1 M NaOH, and the absorbance was measured at 400 nm using a NaOH blank as a reference. The melanin concentration (mg/mL) was calculated using the standard curve equation y = (A_400_ + 0.111) / 0.791. For intracellular melanin, filtered mycelia were dried at 70°C for 48 h, and 0.25 g samples were boiled in 30 mL of 1 M NaOH for 5 h with periodic mixing and volume replenishment. After filtration and dilution to 30 mL with NaOH, absorbance at 400 nm was measured and melanin content (mg/g) was determined as y = (A_400_ + 0.0114)/0.0495.

### Determination of mycotoxin content

Mycotoxins were extracted from *A. alternata* mycelia following an adapted protocol based on Wang et al. [25]. The 0.5 g mycelia obtained from 6 d PDA cultures grown at 28°C in darkness was cryogenically pulverized using liquid nitrogen and transferred to sterile centrifuge tubes. Each sample was homogenized with 2.5 mL of extraction solvent (acetonitrile, water, and formic acid in a volume ratio of 4 : 0.997 : 0.003 (v/v/v)) and agitated at 150 rpm for 30 min at 28°C. Subsequently, 0.25 g anhydrous MgSO_4_ and 0.04 g NaCl were added, followed by vigorous vortexing for 1 min. After centrifugation at 10,000 rpm for 10 min, the supernatant was filtered through a 0.22 μm organic-phase filter and diluted to the final volume of 1.2 mL for liquid chromatography-mass spectrometry (LC-MS) analysis. The Agilent 6460 triple quadrupole mass spectrometer detector is equipped with an electrospray ionization source and operates in positive ionization mode (ESI^+^). Chromatographic quantification was performed against commercial mycotoxin standards, with calibration curves and other HPLC conditions detailed in Table S2 and Table S3.

### The CWDEs activities of cutinase, cellulase, and β-glucosidase assays

The method for determining *A. alternata* CWDEs activity follows Li et al. [18], collecting 0.1 g of mycelium and adding it to the corresponding induction mediums for measurement. Incubate on a shaker at 28°C and 120 rpm, and after3, 6, and 9 d of cultivation, filter the mycelium and collect the culture medium. Then centrifuge at 10,000 rpm for 30 min and take the supernatant as the crude enzyme solution to be tested.

### Pathogenicity assays

The pathogenicity experiment was carried out with reference to the method of Moscoso-Ramírez et al. [26], with slight modifications. Uniformly sized, undamaged Zaosu pear, Huangguan pear, and tomato, surface-sterilized in 0.2 % sodium hypochlorite for 2 min, rinsed thoroughly with distilled water, and air-dried. Three equidistant wounds (3 mm diameter) were made on each fruit using a sterile punch. Each site was inoculated with 20 μL of conidial suspension and air-dried at room temperature for 2 h. By measuring and recording lesion diameters on the 5th and 10th d after inoculation, there were three biological replicates for each condition.

### Y2H assays

Y2H experiments were conducted following Bai et al. [27] with minor adaptations. Full-length AaSlt2 and transcription factors AaSwi6, AaMbp1, and AaRlmA coding sequences were individually cloned into the pGADT7 and pGBKT7 vectors after restriction digestion. All primer sequences used for plasmid construction are provided in Table S1. The resulting plasmid pairs were co-introduced into Y2H Gold, and transformants were initially selected on DDO medium. Protein interactions were assessed by plating on QDO medium, supplemented with X-α-Gal. The pGBKT7-Lam+pGADT7-T and pGBKT7-53+pGADT7-T pairs served as negative and positive controls, respectively.

### RT-qPCR analysis

Total RNA isolation was performed with the TRNzol Universal Reagent (TIANGEN BIOTECH CO., Ltd., Beijing, China, DP424). Reverse transcription of purified RNA into complementary DNA was carried out using the FastKing cDNA First-Strand Synthesis System (TIANGEN BIOTECH CO., Ltd., Beijing, China, KR116). Quantitative PCR assays were subsequently conducted with SuperReal PreMix Plus (SYBR Green) reagents (TIANGEN BIOTECH CO., Ltd., Beijing, China, FP205) on a LightCycler 480 platform (Roche, Germany). The housekeeping gene *GAPDH* served as an endogenous control for normalization, and relative expression levels were determined through the 2^−ΔΔCT^ calculation method. All quantitative primer sequences employed in this study are documented in Supplementary Table S4.

### Statistical analysis

All experimental parameters were replicated at least three times, with results presented as mean values ± standard error (SE). Statistical comparisons were performed using one-way analysis of variance (ANOVA), followed by Duncan’s multiple range test in SPSS 20.0. Significance was defined as *p* < 0.05. Data organization was conducted using Excel 2016.

## Results

### Identification of AaSwi6 and AaMbp1 in *A. alternata*

**Figure 1.**
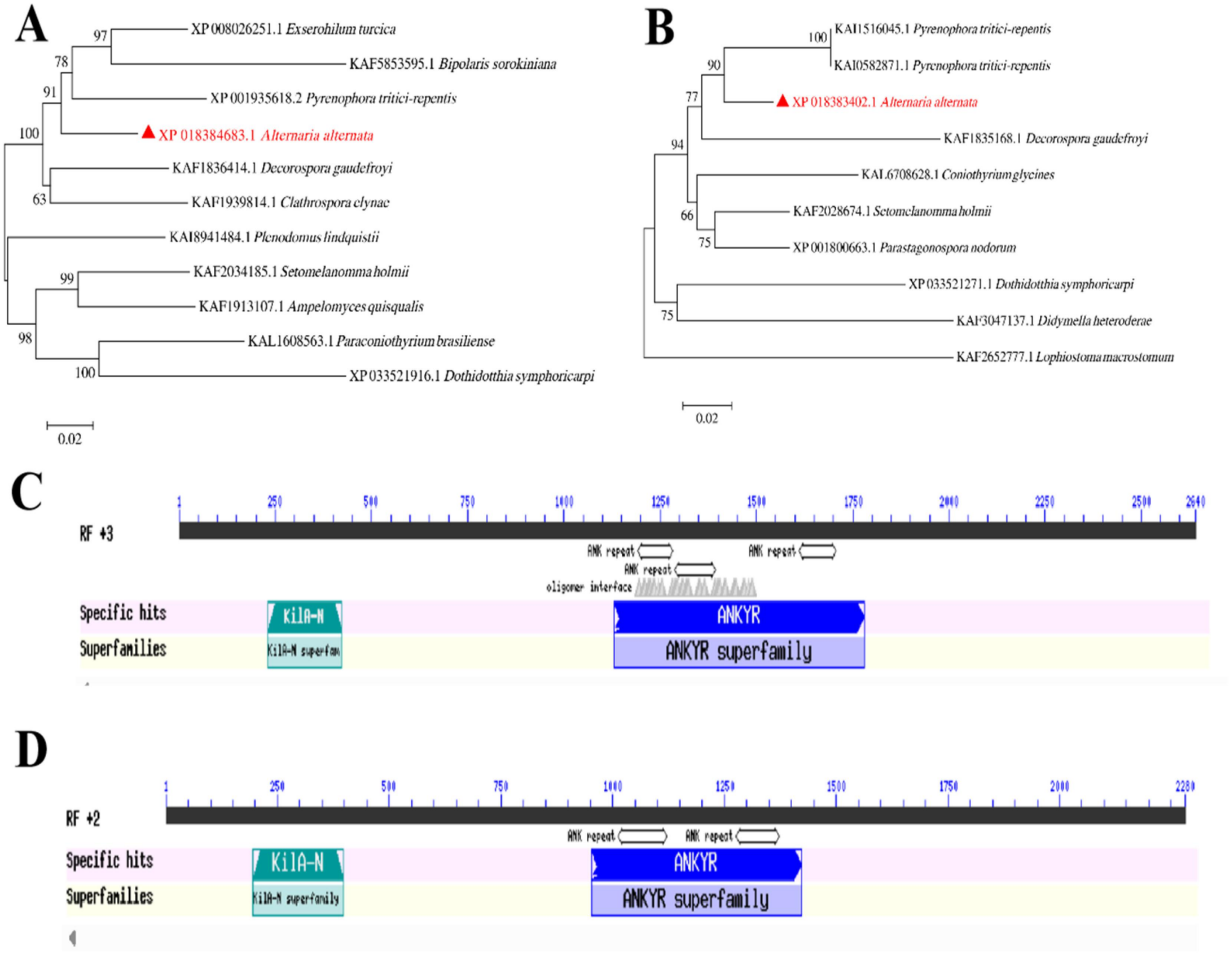
Phylogenetic analysis of transcription factors AaSwi6 (A) and AaMbp1 (B), and conserved domain prediction of AaSwi6 (C) and AaMbp1 (D) in *A. alternata.*

Based on the protein sequences of FvMbp1 and FvSwi6 from *F. verticillioides*, we identified their respective homologs AaMbp1 (29111150) and AaSwi6 (29120169) in *A.alternata* through BLAST analysis. Phylogenetic reconstruction revealed that the AaSwi6 and AaMbp1 proteins from *A.alternata* SRC1lrK2f exhibit the closest evolutionary relationships with their orthologs in *Pyrenophora tritici*-repentis Figure 1(A, B). Examination of conserved domains indicated that both transcription factors belong to members of the ANKYR superfamily and contain the KilA-N structural domain Figure 1(C,D).

### AaMbp1 and AaSwi6 are crucial for the growth and development of *A. alternata*

**Figure 2.**
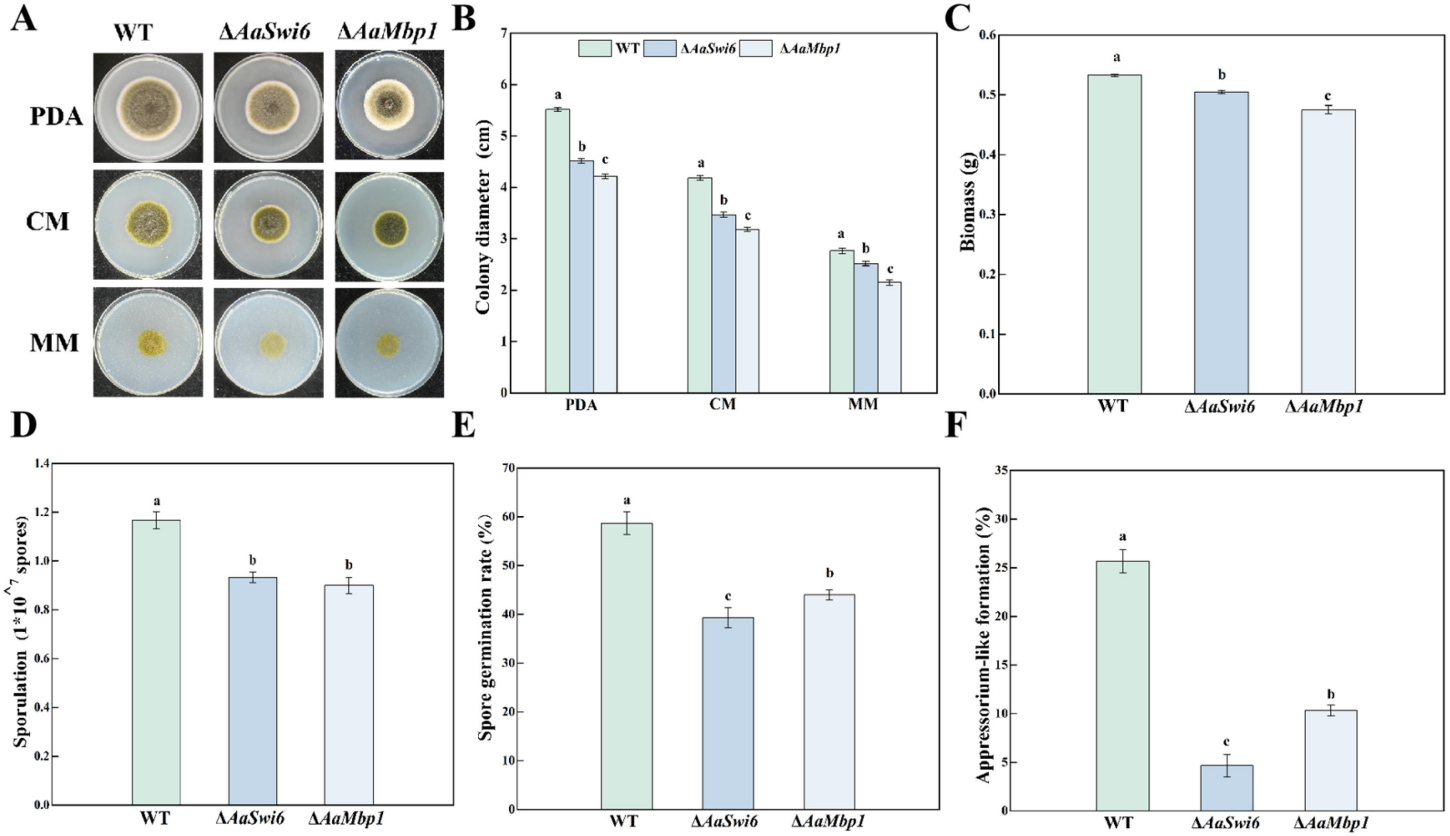
AaSwi6 and AaMbp1 play crucial roles in the growth and developmental processes of *A. alternata*. (A) Vegetative growth of the wild-type (WT), Δ*AaSwi6*, and Δ*AaMbp1* strains on potato dextrose agar (PDA), complete medium (CM), and minimal medium (MM) at 28°C for 5 days. (B) Statistical analysis of WT, Δ*AaSwi6*, and Δ*AaMbp1* strains colony diameters. (C) Statistical analysis of WT, Δ*AaSwi6*, and Δ*AaMbp1* strains biomass. (D) Statistical analysis of WT, Δ*AaSwi6*, and Δ*AaMbp1* strains sporulation. (E) Statistical analysis of WT, Δ*AaSwi6*, and Δ*AaMbp1* strains spore germination. (F) Statistical analysis of WT, Δ*AaSwi6*, and Δ*AaMbp1* strains appressorium-like formation. Different letters indicate significant differences (Duncan’s multiple range test, *P* < 0.05).

To assess the roles of *AaMbp1* and *AaSwi6* in the development of *A.alternata*, we compared the growth characteristics of WT, Δ*AaSwi6*, and Δ*AaMbp1* strains on PDA, MM, and CM medium. Both deletion mutants displayed reduced colony expansion compared to WT, with Δ*AaMbp1* showing more pronounced growth inhibition than Δ*AaSwi6*. Further analysis revealed that biomass accumulation and conidial production were significantly compromised in both mutant strains Figure 2(A–D). To investigate the effects of *AaSwi6* and *AaMbp1* on the differentiation of infection structures in *A. alternata*, we monitored conidial germination and appressorium-like formation on hydrophobic surfaces. After 3 h of incubation, Δ*AaSwi6* and Δ*AaMbp1* displayed impaired germination efficiency, with Δ*AaSwi6* exhibiting more severe defects. Following 6 h of cultivation, both mutants displayed reduced rates of appressorium-like differentiation, again with Δ*AaSwi6* demonstrating stronger phenotypic effects Figure 2(E–F).

### AaMbp1 and AaSwi6 are involved in the stress response of *A. alternata*

**Figure 3.**
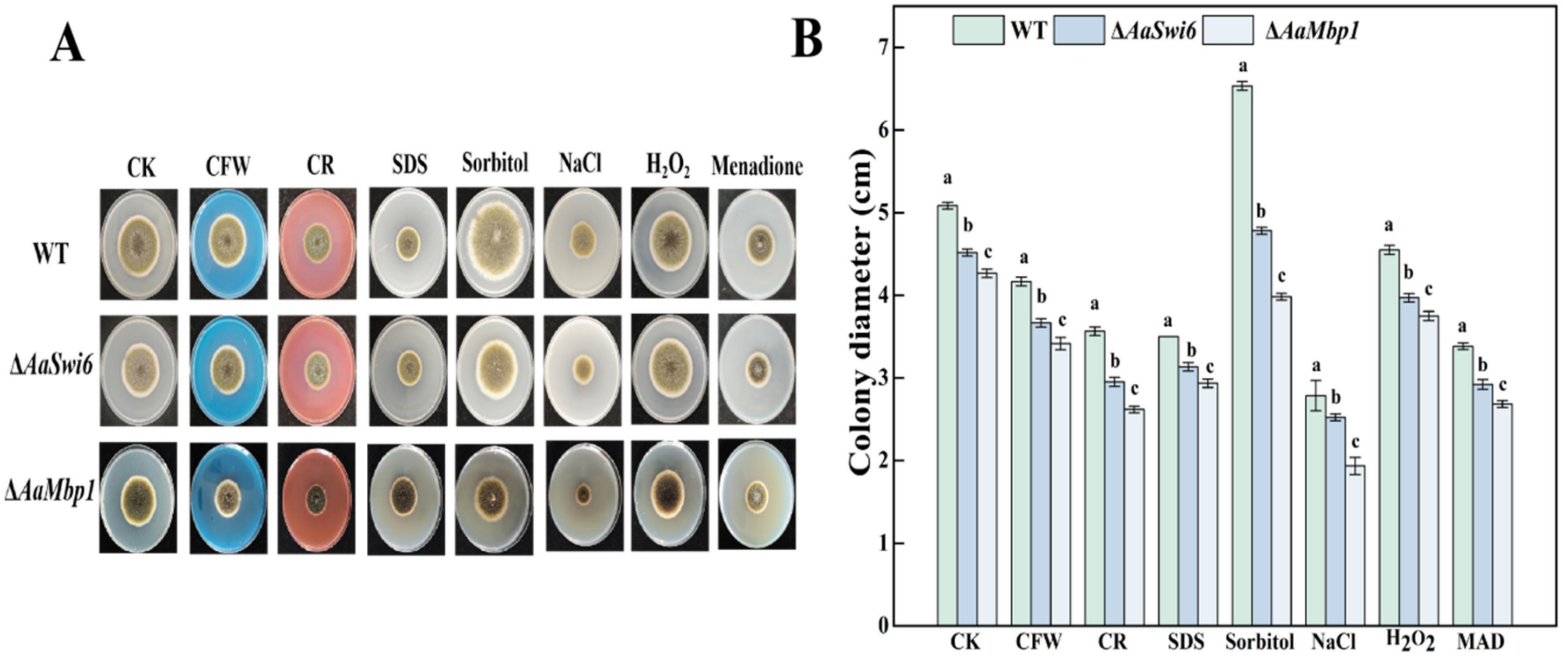
AaSwi6 and AaMbp1 are involved in the stress response of *A. alternata*. (A) vegetative growth of WT, Δ*AaSwi6*, and Δ*AaMbp1* strains on PDA medium supplemented with 50 μg/mL calcofluor white (CFW), 200 uM cong red (CR), 0.02% sodium dodecyl sulfate (SDS), 1 M sorbitol, 1 M NaCl, 5 mM H_2_O_2_ and 60 uM menadione (MAD) at 28°C for 5 days. (B) statistical analysis of colony diameters of WT, Δ*AaSwi6*, and Δ*AaMbp1* strains under different exogenous stress. Different letters indicate significant differences (Duncan’s multiple range test, *p* < 0.05).

To evaluate the stress response characteristics of the mutants, we cultured each strain under different stress conditions. Both Δ*AaSwi6* and Δ*AaMbp1* showed slightly increased sensitivity to cell wall stressors, with Δ*AaMbp1* exhibiting the highest inhibition rates for Congo red (38.64%), CFW (19.91%), and SDS (31.38%), all exceeding those of WT (29.72%, 17.91%, and 31.10%, respectively) and Δ*AaSwi6* (34.73%, 18.81%, and 30.75%, respectively). Under osmotic stress, Δ*AaMbp1* demonstrated the highest inhibition rates for NaCl (54.8%) and sorbitol (6.8%), while WT showed inhibition rates of 45.28% and −28.54% to NaCl and sorbitol respectively, and Δ*AaSwi6* showed 44.47% and −5.75% respectively. Under oxidative stress, Δ*AaMbp1* displayed the highest inhibition rates for H_2_O_2_ (12.18%) and MAD (38.41%), compared to WT (10.43% and 33.46%, respectively) and Δ*AaSwi6* (12.17% and 35.4%, respectively). These results collectively demonstrate that *AaSwi6* and *AaMbp1* play crucial roles in maintaining cellular homeostasis under various stress conditions Figure 3(A–B).

### AaMbp1 and AaSwi6 are involved in the cell wall composition of *A. alternata*

**Figure 4.**
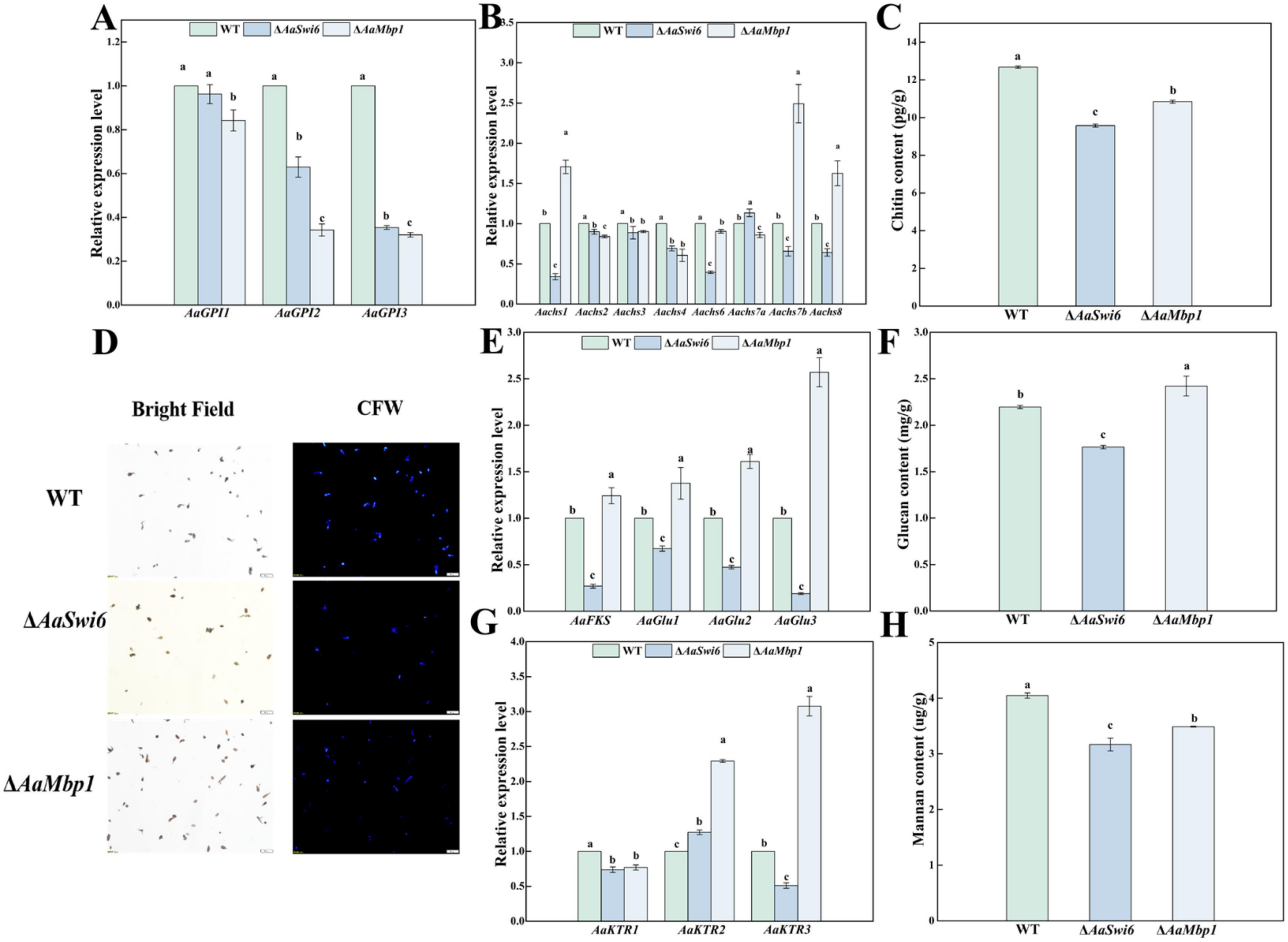
AaSwi6 and AaMbp1 are necessary for the cell wall compositions of *A. alternata*. (A) Expression analyses of genes related to GPI-anchored proteins. (B) expression analyses of genes related to chitin synthase. (C) the chitin content of WT, Δ*AaSwi6*, and Δ*AaMbp1* strains on PDA at 28°C for 6 d. (D) CFW staining of WT, Δ*AaSwi6*, and Δ*AaMbp1* spores. (E) expression analyses of genes related to glucan synthase. (F) the glucan content of WT, Δ*AaSwi6*, and Δ*AaMbp1* strains on PDA at 28°C for 6 d. (G) expression analyses of genes related to mannan synthase. (H) the mannan content of WT, Δ*AaSwi6*, and Δ*AaMbp1* strains on PDA at 28°C for 6 d. Different letters indicate significant differences (Duncan’s multiple range test, *p* < 0.05).

GPI-anchored proteins play a crucial role in the biosynthesis and organization of fungal cell wall polysaccharides [28]. In our study, the disruption of *AaSwi6* and *AaMbp1* resulted in a significant downregulation of transcription for the genes encoding GPI-anchored proteins *AaGPI1*, *AaGPI2*, and *AaGPI3* Figure 4(A). Analysis of chitin synthase expression revealed distinct regulatory patterns: the deletion of *AaSwi6* suppressed the transcription of *Aachs1*, *Aachs2*, *Aachs3*, *Aachs4*, *Aachs6*, *Aachs7b*, and *Aachs8*, whereas the disruption of *AaMbp1* downregulated *Aachs2*, *Aachs3*, *Aachs4*, *Aachs6*, and *Aachs7a*, while upregulating *Aachs1*, *Aachs7b*, and *Aachs8*. Quantitative analysis of chitin content indicated that the chitin levels in the mutant strains Δ*AaSwi6* and Δ*AaMbp1* decreased by 24.47% and 14.44%, respectively. Observation following staining with CFW reagent demonstrated that the fluorescence intensity of the WT was significantly stronger than that of Δ*AaSwi6* and Δ*AaMbp1*, with Δ*AaMbp1* exhibiting a fluorescence intensity that was notably stronger than that of Δ*AaSwi6* Figure 4(B–D).

In comparison to the WT, the deletion of *AaSwi6* led to a downregulation of the glucan synthesis genes *AaFKS*, *AaGlu1*, *AaGlu2*, and *AaGlu3*. Conversely, the deletion of *AaMbp1* resulted in an upregulation of *AaGlu1*, *AaGlu2*, and *AaGlu3*, while having no effect on the expression of *AaFKS*. Quantitative analysis revealed that the glucan content in the Δ*AaSwi6* strain decreased by 20 % compared to WT whereas the glucan content in the Δ*AaMbp1* strain increased by 10.91 %. This suggests that the deletion of *AaSwi6* has a greater impact on the glucan content of cell wall components Figure 4(E–F). Additionally, the deletion of *AaSwi6* led to downregulation of mannan synthesis genes *AaKTR1*, *AaKTR2*, and *AaKTR3*, while the deletion of *AaMbp1* resulted in the downregulation of *AaKTR1* only, with the other two mannan synthesis genes being upregulated. Quantitative analysis revealed that the mannan levels in the Δ*AaSwi6* and Δ*AaMbp1* strains decreased by 21.73 % and 13.83 %, respectively, further indicating that the deletion of *AaSwi6* has a more significant impact on the mannan content of the cell wall components Figure 4(G–H).

### AaMbp1 and AaSwi6 are both involved in the production of secondary metabolites in *A. alternata*

**Figure 5.**
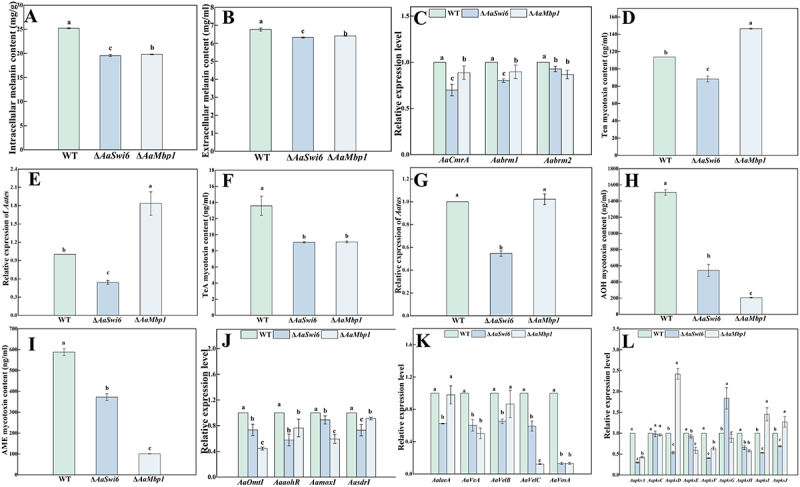
AaSwi6 and AaMbp1 are involved in the production of secondary metabolites of *A. alternata*. (A-B) the intracellular and extracellular melanin content of WT, Δ*AaSwi6*, and Δ*AaMbp1* strains on PDB at 28°C for 6 d, (C) expression analyses of genes related to DHN melanin synthase, (D-J) the mycotoxin content and related gene expression of WT, Δ*AaSwi6*, and Δ*AaMbp1* strains on PDA at 28°C for 6 d, (D) Ten toxin content, (E) Ten toxin synthase-related gene expression, (F) TeA toxin content, (G) TeA toxin synthase-related gene expression, (H) AOH toxin content, (I) AME toxin content, (J) AOH and AME toxin synthase-related genes expression, (K) expression analyses of genes related to Velvet complex synthase, (L) expression analyses of genes related to polyketide synthase. Different letters indicate significant differences (Duncan’s multiple range test, *p* < 0.05).

To examine the involvement of *AaMbp1* and *AaSwi6* in secondary metabolism, we quantified melanin production in WT and mutant strains. Both intracellular and extracellular melanin accumulation were significantly reduced in Δ*AaSwi6* and Δ*AaMbp1*, which was accompanied by the downregulation of key transcription factor and genes involved in melanin biosynthesis. Notably, the loss of *AaSwi6* had an even greater impact on melanin synthesis Figure 5(A–C).

To further explore the effects of *AaMbp1* and *AaSwi6* on the synthesis of secondary metabolites, specifically the mycotoxins of *A. alternata*, we extracted and measured the mycotoxins from WT, Δ*AaSwi6*, and Δ*AaMbp1* strains. The results indicated that, compared to the WT, the Δ*AaSwi6* strain exhibited a decrease in the contents of nHST mycotoxins TeA, Ten, AOH, and AME by 33.28%, 22.29%, 63.98%, and 36.71%, respectively. In contrast, the Δ*AaMbp1* strain showed the reduction in the contents of TeA, AOH, and AME decreased by 33%, 86.32%, and 82.85%, while the content of Ten increased by 28.89%. The Δ*AaSwi6* strain showed coordinated downregulation of key biosynthetic genes, including *Aatas1*, *Aates*, *AaaohR*, *AaOmtI, AasdrI*, and *AaMoxI*. Conversely, in Δ*AaMbp1*, the *Aates* gene was upregulated, while *Aatas1*, *AaaohR*, *AaOmtI*, *AasdrI*, and *AaMoxI*, which are responsible for AOH and AME toxin synthesis were all downregulated Figure 5(D–J).

Velvet proteins form multiple complexes that collectively regulate fungal growth, development, and secondary metabolism [29]. In the Δ*AaSwi6* strain, all Velvet genes (*AalaeA, AaVeA, AaVelB, AaVelC*, and *AaVosA)* were downregulated. In contrast, the Δ*AaMbp1* strain exhibited downregulation of only *AaVeA*, *AaVelC*, and *AaVosA*, while the remaining genes maintained expression levels comparable to those of the wild type Figure 5(K).

Polyketide synthases (PKS) encode critical enzymes that facilitate the synthesis of polyketide and directly regulate the production of secondary metabolites [30]. Notably, *AapksA* participates in the synthesis of both DHN melanin and toxin, while *AapksI, AapksJ, AapksH*, and *AapksF* primarily involved in toxin biosynthesis. Among the ten PKS genes identified in *A.alternata*, expression analysis was conducted on all genes except *AapksB*, which showed negligible expression in previous transcriptomic studies. In the Δ*AaSwi6* strain, seven PKS genes (*AapksA, AapksD, AapksE, AapksF, AapksH, AapksI*, and *AapksJ*) were found to be downregulated. Conversely, the Δ*AaMbp1* strain displayed a different expression pattern, with only five PKS genes (*AapksA, AapksE, AapksF, AapksG*, and *AapksH*) exhibiting reduced expression Figure 5(L).

### Both AaMbp1 and AaSwi6 are involved in the pathogenicity of *A. alternata*

**Figure 6.**
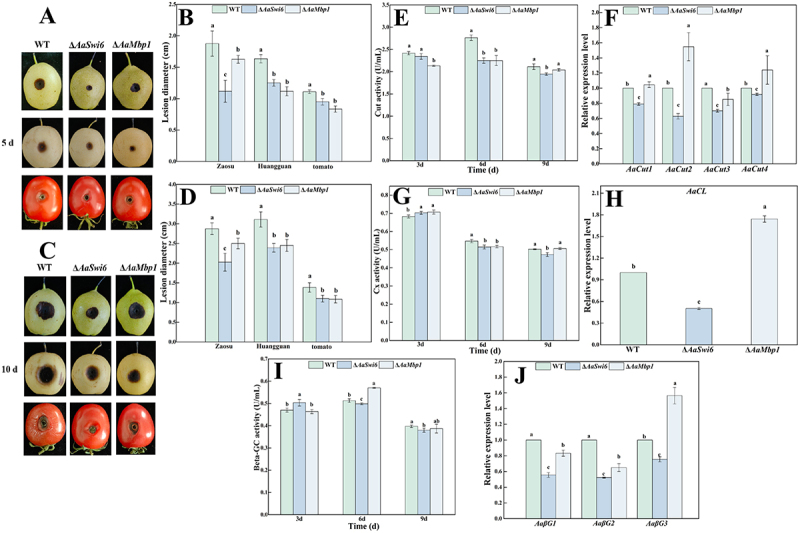
Both AaSwi6 and AaMbp1 are involved in the pathogenicity of *A. alternata*. (A-D) after inoculating “Zaosu” pear, “Huangguan” pear, and tomato with WT, Δ*AaSwi6*, and Δ*AaMbp1* strains, the lesion diameters were measured. (A) 5 d lesion morphology, (B) 5 d lesion diameter, (C) 10 d lesion morphology, (D) 10 d lesion diameter, (E) Cutinase (cut) activity of WT, Δ*AaSwi6*, and Δ*AaMbp1* strains on 3, 6, and 9 d, (F) Expression analyses of genes related to cutinase synthase, (G) Cellulase (CX) activity of WT, Δ*AaSwi6*, and Δ*AaMbp1* strains on 3, 6, and 9d, (H) Expression analyses of genes related to cellulase synthase, (I) β-glucosidase (beta-GC) activity of WT, Δ*AaSwi6*, and Δ*AaMbp1* strains on 3, 6, and 9d, (J) Expression analyses of genes related to β-glucosidase synthase. Different letters indicate significant differences (Duncan’s multiple range test, *p* < 0.05).

Pathogenicity assays conducted on multiple host plants revealed a consistent reduction in lesion development for both mutant strains. At 5 d post-inoculation, Δ*AaSwi6* and Δ*AaMbp1* showed lesion areas that were reduced by 40.43 % and 13.3 % respectively on Zaosu pear; 23.31 % and 31.29 % on Huangguan pear, and 14.41 % and 25.26 % on tomato when compared to the WT. By 10 d post-inoculation, the disease development remained attenuated, with Δ*AaSwi6* and Δ*AaMbp1* exhibiting 30.56 % and 13.2 % smaller lesions on Zaosu pear; 23.15 % and 21.11 % on Huangguan pear, and 20.29 % and 21.74 % on tomato relative to the controls Figure 6(A–D). *Cut*, *Cx*, and *BG* are key genes encoding CWDEs. In Δ*AaSwi6*, the expression of *Cut*, *Cx*, and *BG* was downregulated; however, in Δ*AaMbp1*, the regulatory effect on these genes was not significant. Further measurements of enzyme activity at 3, 6, and 9 d revealed that the activities of these CWDEs were lower in Δ*AaSwi6* than in WT. In contrast, the impact of Δ*AaMbp1* on these enzymatic activities was less pronounced Figure 6(E–J).

### The upstream kinase AaSlt2 of A. alternata is involved in regulating AaSwi6, rather than AaMbp1

**Figure 7.**
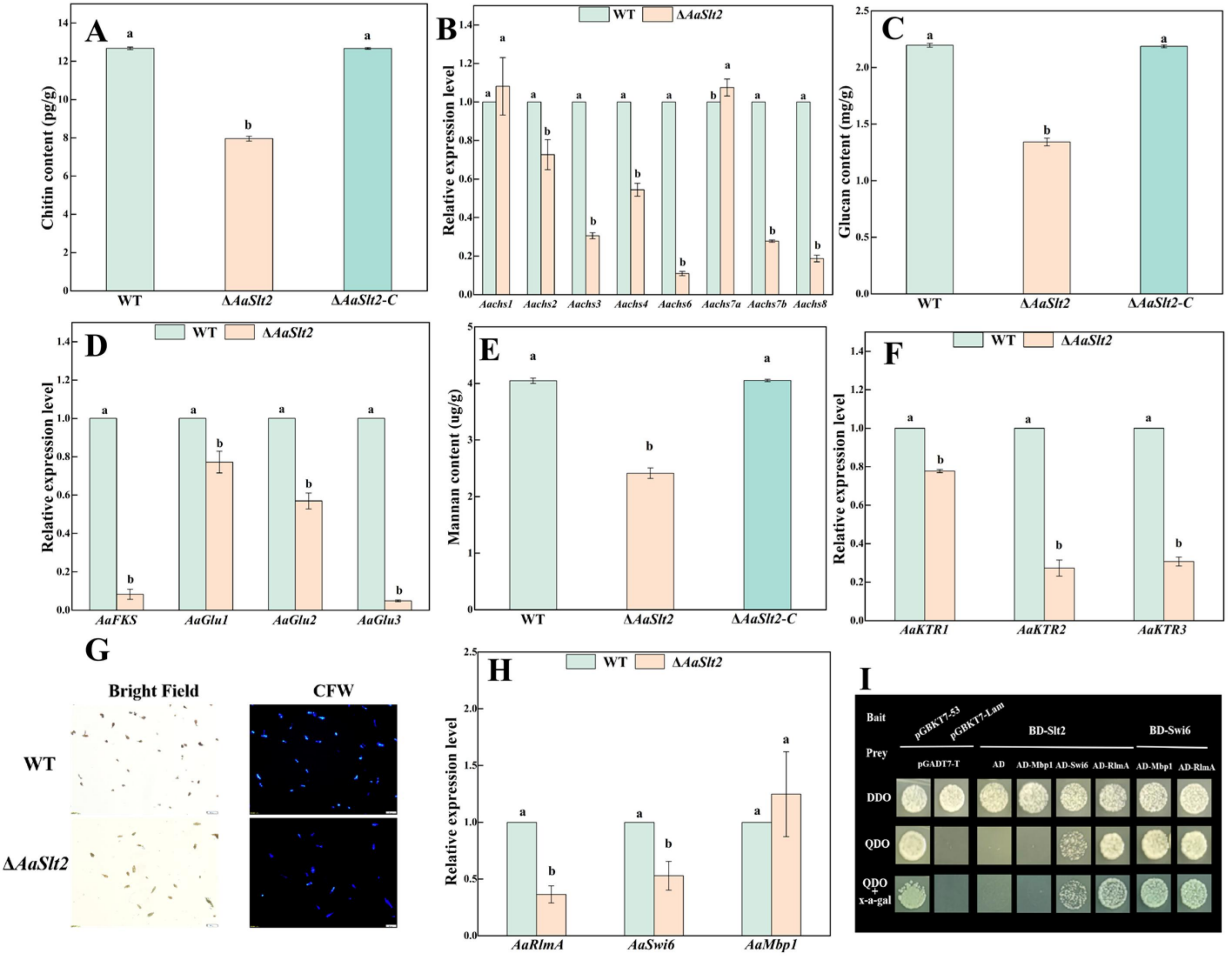
AaSlt2 is necessary in the cell wall compositions of *A. alternata*. (A) The chitin content of WT, Δ*AaSlt2* and Δ*AaSlt2-C* strains on PDA at 28°C for 6 d, (B) Expression analyses of genes related to chitin synthase, (C) The glucan content of WT, Δ*AaSlt2* and Δ*AaSlt2-C* strains on PDA at 28°C for 6 d, (D) Expression analyses of genes related to glucan synthase, (E) The mannan content of WT, Δ*AaSlt2*, and Δ*AaSlt2-C* strains on PDA at 28°C for 6 d, (F) Expression analyses of genes related to mannan synthase, (G) CFW staining of WT, Δ*AaSlt2*, and Δ*AaSlt2-C* spores, (H) Expression analyses of *AaRlmA, AaSwi6* and *AaMbp1* in WT and Δ*AaSlt2*, (I) Y2H was used to verify the interactions between AaSlt2 and AaRlmA and AaSwi6, as well as the interactions between AaSwi6 and AaRlmA and AaMbp1 at 5d. DDO represents SD medium lacking trp and leu, QDO represents SD medium lacking trp, leu, his, and Ade. pGBKT7-53/pGADT7-T was used as the positive control, and pGBKT7-Lam/pGADT7-T as the negative control.

Due to the distinct in biological functions of Δ*AaMbp1* and Δ*AaSwi6*, the Δ*AaSwi6* strain induced more significant alterations in the cell wall component content of *A. alternata* compared to Δ*AaMbp1*. Given considering our previous finding that the MAPK kinase AaSlt2 in the CWI signaling pathway is highly sensitive to cell wall stress, we further quantified cell wall components and the expression of related genes in the Δ*AaSlt2* mutant strain, and the Δ*AaSlt2* mutant strain was derived from our previous construction [31]. Compared to the WT, deletion of *AaSlt2* resulted in downregulation of chitin synthesis genes (*Aachs2-Aachs6*, *Aachs7b*, and *Aachs8*), glucan synthesis genes (*AaFKS*, *AaGlu1*, *AaGlu2*, and *AaGlu3*), and mannan synthesis genes (*AaKTR1*, *AaKTR2*, and *AaKTR3*). The contents of chitin, glucan, and mannan decreased by 37.17 %, 39.09 %, and 40.49 %, respectively. Following CFW staining, the WT exhibited significantly stronger fluorescence intensity than the Δ*AaSlt2* strain. The results indicated that the deletion of the *AaSlt2* gene led to a decrease in the levels of three cell wall components: chitin, glucan, and mannan (Figure 7(A-G). While deletion of the *AaSwi6* and *AaMbp1* also resulted in alterations to the cell wall components, it is noteworthy that both *AaSlt2* and *AaSwi6* deletions resulted in a reduction of the three primary cell wall components. This suggests that these genes may play a more comparable role in the regulation of cell wall synthesis.

Compared to WT, both *AaSwi6* and *AaRlmA* were downregulated in Δ*AaSlt2*, while the expression levels of *AaMbp1* remained unaffected by the deletion of *AaSlt2* Figure 7(H). Using Y2H assays, we found that Y2H strains harboring AaSlt2-BD could grow on QDO medium supplemented with X-α-gal and turned blue when paired with AaSwi6-AD and AaRlmA-AD. Conversely, Y2H strains containing AaMbp1-AD were unable to grow on QDO medium with X-α-gal, indicating that AaSlt2 physically interacts with AaSwi6 and AaRlmA but does not physically interact with AaMbp1 Figure 7(I). We further employed Y2H to analyze the interactions among multiple transcription factors downstream of the CWI signaling pathway, revealing that AaSwi6-BD also interacts with AaMbp1-AD and AaRlmA-AD. Strains with AaSwi6-BD grew and turned blue on X-α-gal-supplemented QDO medium, confirming that AaSwi6 physically binds to both AaMbp1 and AaRlmA, likely performing specific biological roles through these interactions Figure 7(I). The above experimental results elucidate why deletion of *AaSwi6* does not severely damage the cell wall composition. This is due to the fact that, in addition to AaSwi6, AaRlmA is also a transcription factor directly regulated downstream of AaSlt2, and AaSwi6 itself can interact with AaRlmA. In our previous work, we constructed Δ*AaRLMA* and found that, compared with WT, its cell wall components were also affected [18].

## Discussion

The conserved CWI MAPK signaling cascade regulates fungal growth, stress adaptation, secondary metabolism, and pathogenicity [11]. Within this pathway, the downstream kinase Slt2 modulates transcription factors such as the MBF complex and RlmA, which in turn induce the expression of genes involved in cell wall biosynthesis and pathogenicity [32]. In this study, we individually knocked out MBF complex genes *AaSwi6* and *AaMbp1*, revealing that they jointly participate in regulating the growth, development, and pathogenicity of *A. alternata*, while differentially regulating cell wall synthesis and secondary metabolite production.

In fungi, Swi6 has been more extensively characterized compared to Mbp1. Both proteins exhibit a conserved architecture that includes a KilA-N domain and several ANK repeats [19]. As members of the APSES family within the bHLH (basic helix-loop-helix) class, these transcription factors are known to perform fundamental regulatory functions in fungi, modulating processes such as growth and development, morphogenesis, stress adaptation, and virulence [33–35]. Our research indicates that both AaSwi6 and AaMbp1 possess one KilA-N domain and multiple ANK domains Figure (1). In *F. verticillioides*, the domains of FvMbp1 and FvSwi6 proteins consist of an N-terminal APSES-type DNA-binding domain and two ANK domains [19]. Similarly, CfSwi6 from *C.fimbriata* also possesses a fungal-specific APSES-type DNA-binding domain [36], underscoring the structural conservation of these regulatory proteins across fungi.

Our research results indicate that AaSwi6 and AaMbp1 play crucial roles in regulating the growth, development, and infectivity of *A. alternata*. Compared to the WT, the mutant strains lacking these two transcription factors exhibited significantly slower colony growth rates, alongside suppressed spore production, biomass accumulation, and infectivity Figure (2). In *A. oligospora*, the deletion of *AoSwi6* significantly impairs mycelial growth, alters mycelia morphology, disrupts sporulation capability, downregulates the expression of sporulation-related genes, and reduces spore germination rates [21]. Similarly, in *Pleurotus ostreatus*, the deletion of *Mbp1* resulted in slower colony growth, reduced mycelial biomass, and shorter aerial hyphae [37]. Furthermore, the rice blast fungus *M.oryzae* also exhibited growth defects and abnormal hyphal and appressorial morphology upon disruption of *MoSwi6* [38]. The deletion of these two transcription factors affects the growth and development of *A. alternata*. Although the deletion of *AaMbp1* has a more significant impact on the colony growth rate than the deletion of *AaSwi6*, the loss of *AaSwi6* has a more pronounced effect on cell wall integrity compared to the deletion of *AaMbp1*. Therefore, we preliminarily propose that the reason why infection structure differentiation in Δ*AaSwi6* occurs at a slower rate than in Δ*AaMbp1* may be related to the damage to cell wall integrity, although the underlying molecular mechanisms require further investigation.

The cell wall is crucial for key life activities of fungi, including maintaining their morphology, growth and development, environmental adaptation, and pathogenicity. The fungal cell wall can undergo dynamic remodeling, enabling a rapid response to stress through structural adjustments, which aids fungi in surviving adverse environments [39]. The cell wall of *A. alternata* is mainly responsible for synthesizing cell wall components such as chitin, glucan, and mannan facilitated by genes including *AaGPI1-3*, *Aachs1-8*, *AaFKS*, *AaGlu1-3*, and *AaKTR1-3*. Our research results indicate that deletion of *AaSwi6* and *AaMbp1* leads to slight changes in sensitivity to external stress Figure (3). Additionally, compared to the WT, the contents of chitin, glucan, and mannan decreased in Δ*AaSwi6*, whereas in Δ*AaMbp1*, only the chitin and mannan content were reduced, and although the deletion of *AaMbp1* results in a decrease in chitin content, the expression of chitin synthesis‑related genes *Aachs1*, *Aachs7b*, and *Aachs8* is upregulated. This observation suggests that the deletion of *AaMbp1*, which hinders chitin synthesis, prompts *A. alternata* to activate a compensatory mechanism aimed at restoring chitin synthesis or mitigating damage by upregulating the expression of other related genes. Furthermore, the deletion of *AaMbp1* is associated with an increase in glucan content Figure (4), potentially due to alterations in the composition and structure of the cell wall, which may lead to the upregulation of certain related genes to compensate for cell wall defects. In *F. verticillioides*, deletion of *FvMbp1* and *FvSwi6* results in higher tolerance to exogenous stress agents [19]. In *C. fimbriata*, deletion of *CfSwi6* leads to increased sensitivity to osmotic stress but enhanced tolerance to cell wall stress, alongside decreased levels of chitin, β-glucan, and mannan [36]. In *P. ostreatus*, the deletion of *mbp1* resulted in a thinner cell wall, impaired β-glucan synthesis, and affected the expression of chitin synthase genes [36]. In *M. oryzae*, the deletion of *MoSwi6* increases resistance to cell wall inhibitors and sorbitol [37]. These results indicate that these two APSES transcription factors are indeed related to cell wall integrity in fungi; however the reason for the differences in stress response may be attributed to strain specificity.

1,8-Dihydroxynaphthalene (DHN) melanin is a crucial secondary metabolite in *A.alternata*, primarily localized within the hyphal and spore cell walls, where it plays a significant role in fungal development and virulence [40]. Its biosynthesis is regulated by the transcription factor *AaCmrA*, while the genes *AapksA*, *Aabrm1*, and *Aabrm2* are directly involved in the synthesis of DHN melanin [22,24,41,42]. We observed that both Δ*AaSwi6* and Δ*AaMbp1* exhibited reduced intracellular and extracellular melanin accumulation compared to the WT, which correlates with the downregulated expression of *AaCmrA, Aabrm1,* and *Aabrm2* Figure 5(A-C). In *M. oryzae*, the deletion of *MoSwi6* leads to reduced deposition of DHN melanin [38]. In *Aureobasidium melanogenum*, the deletion of *Swi4* results in a reduction in melanin content and downregulation of melanin synthesis transcription factors and related genes [43]. During the infection of plants by *A. alternata*, ATs serve as important secondary metabolites, acting as virulence and colonization factors closely associated with pathogenicity. Among these, TeA, AME, and AOH are fungal toxins that are widely found postharvest [3]. TeA synthetase (TAS1) is responsible for driving TeA production [44], while the biosynthesis of AOH and AME involves OmtI, moxI, sdrI, and aohR [45]. The synthesis pathway for Ten is modulated by the *tes* gene [46]. Our research indicates that compared to WT, the levels of TeA, Ten, AOH, and AME toxins are significantly reduced in Δ*AaSwi6*, while in Δ*AaMbp1*, the levels of TeA, AOH, and AME toxins are decreased, although the level of TeA toxin shows an increase. The expression trends of the genes related to the synthesis of these toxins are also generally align with the toxin content results Figure 5(D-J). In *A. alternata*, the deletion of the CWI MAPK gene *AaSlt2* resulted in decreased levels of fungal toxins ALT, Ten, and ACT [16,31]; similarly, the deletion of transcription factor *AaRlmA* resulted in reduced levels of fungal toxins Ten, TeA, AOH, and AME [18]. In *F. verticillioides*, knocking out the MAPKKK gene *FvBck1* significantly reduced the accumulation of fumonisin B1 toxin [47]. CWDEs are essential for pathogenicity. Our results indicate that the expression of *Cut*, *Cx*, and *BG* is downregulated in both Δ*AaSwi6* and Δ*AaMbp1* (Figure 6). In *F. verticilloides*, the deletion of *FvMbp1* and *FvSwi6* results in significant downregulation of multiple cell wall-degrading enzyme genes [19].

**Figure 8.**
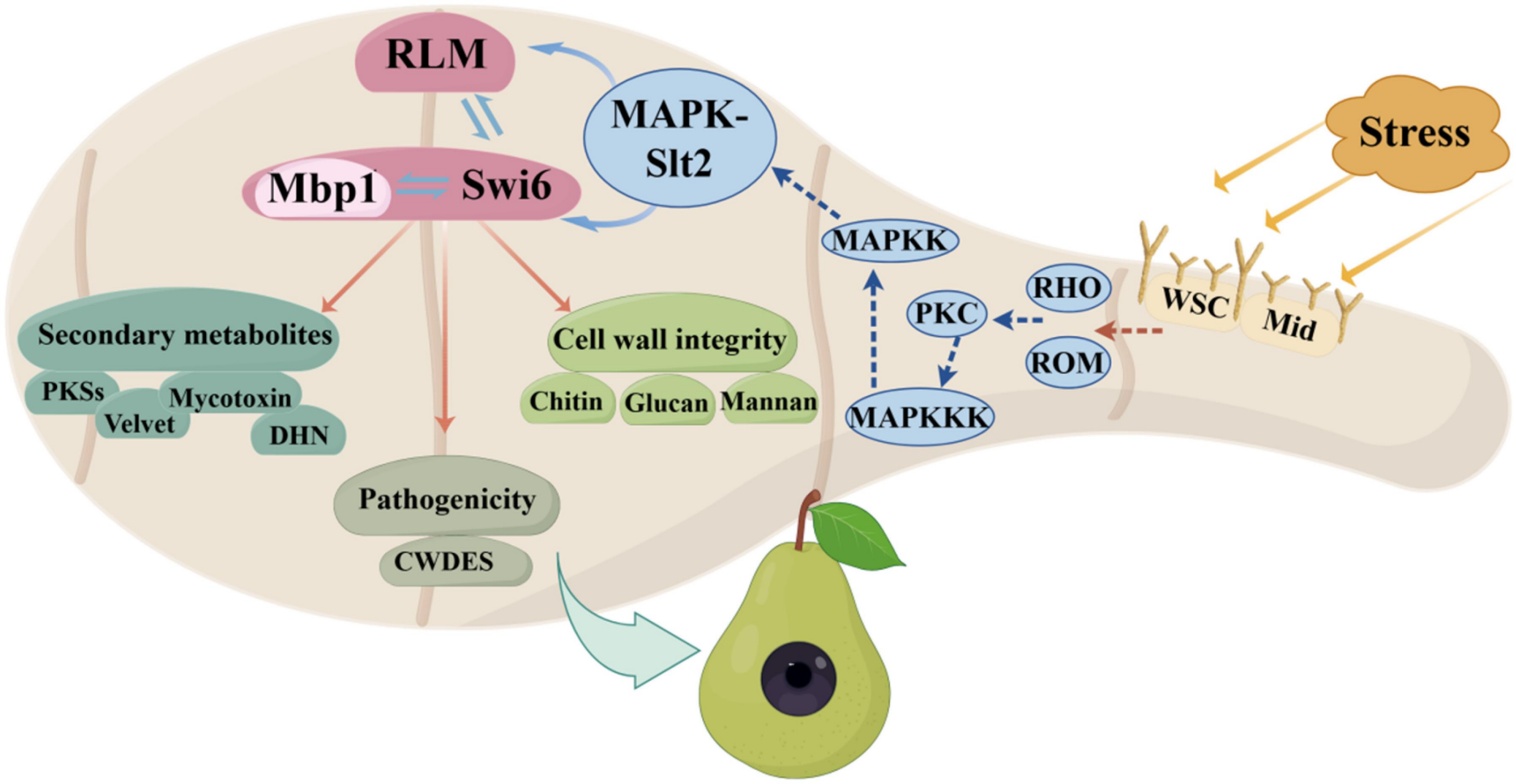
The regulatory model of signal pathway CWI MAPK AaSlt2-AaSwi6 on cell wall integrity, secondary metabolites production and pathogenicity in *A.Alternata*.

Our research results indicate that, compared to the WT, the cell wall components chitin, glucan, and mannan in Δ*AaSlt2* are reduced, with the degree of reduction being less pronounced than that observed in Δ*AaSwi6* and Δ*AaMbp1*. The expression of related cell wall synthesis genes is downregulated, including *AaRlmA* and *AaSwi6*, while no regulatory effect on the expression of *AaMbp1* is observed. Y2H results further demonstrate found that AaSlt2 interacts with downstream transcription factors AaSwi6 and AaRlmA, but does not interact with AaMbp1, thereby regulating cell wall synthesis. Notably, AaSwi6 can interact with both AaMbp1 and AaRlmA, allowing it to perform specific functions (Figure 7). In *F. verticillioides*, FvMbp1 interacts with FvSwi6 to form a complex that regulates vegetative growth, stress responses, and pathogenicity [19]. Similarly, in *A. oligospora*, both AoPKC and AoSlt2 interact with AoSwi6 to regulate asexual development, cell wall integrity, stress responses, and lifestyle transitions [21]. In *C. fimbriata*, the interaction between CfSlt2 and CfSwi6 revealed the molecular mechanism by which the CWI-MAPK pathway regulates pathogenicity through CfSwi6-mediated downstream gene regulation [48]. In *Beauveria bassiana*, the transcriptional activator Swi6 interacts with Mbp1 to regulate hyphal differentiation and virulence [49]. Therefore, AaSwi6 and AaMbp1 are pivotal in the pathogenicity of *A. alternata*. Beyond affecting growth and development, secondary metabolite production, and CWDEs, they also regulate cell wall synthesis by activating AaSwi6 expression through the upstream MAPK kinase AaSlt2. This study suggests that AaSwi6 may be directly regulated by AaSlt2, playing a key role in the regulation of growth and development, cell wall synthesis, secondary metabolite production, and pathogenicity in *A. alternata*. Although transcription factor AaMbp1 was not directly regulated by AaSlt2, it interacted with AaSwi6 and also played a crucial role in regulating growth and development, secondary metabolite synthesis, and pathogenicity in *A. alternata* (Figure 8). This study elucidates the potential regulatory network of downstream transcription factors within the CWI MAPK pathway, thereby enriching the understanding of the signaling mechanisms involved in postharvest pathogenic fungi. Future research may further elucidate the molecular regulatory mechanisms of downstream transcription factors in the CWI MAPK signaling pathway concerning specific cell wall components, thereby constructing a broader regulatory network that provides theoretical support for a deeper understanding of fungal biology and the development of innovative disease control technologies.

## Supplementary Material

Supplemental Material

## Data Availability

All datasets generated for this study are included in this article and the supplementary files. The data that support the findings of this study are openly available in figshare (https://doi.org/10.6084/m9.figshare.31963173) .
