# Supplementary material for: The Slt2-Swi6 signaling pathway plays a pivotal role in regulating cell wall biosynthesis, secondary metabolite production, and the pathogenicity of pear fungal A. alternata
Source: Virulence. 2026 Aug 5;17(1):2715208. doi: 10.1080/21505594.2026.2715208 (PMC13479823; doi:10.1080/21505594.2026.2715208)
Supplement: Supplementary file 1 [file KVIR_A_2715208_SM5959.docx]

**Table S1 Primers used for vector constructions**

| Gene | Primer sequences (5' - 3') |
| --- | --- |
| *AaSwi6-F* | ATGTACGCCCAATCTCCCCAT |
| *AaSwi6-R* | TTACTCTACGCCCTCAACCTT |
| *AaMbp1-F* | ATGCCCCCCGCGCCCGATGGCAAA |
| *AaMbp1-R* | AACAAAACCTATAGCGGACT |
| *AaSwi6-up-F* | GCACGCAACCGAGAATAG |
| *AaSwi6-up-R* | TTGACCTCCACTAGCTCCAGCCAAGCCGGGAAGGATAGCGACGAG |
| *AaSwi6-down-F* | GAATAGAGTAGATGCCGACCGCGGGTTAGTAGGTCGGGTCAGGGAG |
| *AaSwi6-down-R* | TGGACAAGGTGGCAAAGG |
| *AaMbp1-up-F* | GTGCGACTCCAGAAACCG |
| *AaMbp1-up-R* | TTGACCTCCACTAGCTCCAGCCAAGCCCGACCGCAAGTGAACCTC |
| *AaMbp1-down-F* | GAATAGAGTAGATGCCGACCGCGGGTTGACCAAGATAGCGAGCATT |
| *AaMbp1-down-R* | TGTTCATCCCAGTCGTCAA |
| *H1-F* | GGCTTGGCTGGAGCTAGTGGAGGTCAA |
| *H1-R* | GTATTGACCGATTCCTTGCGGTCCGAA |
| *H2-F* | GATGTAGGAGGGCGTGGATATGTCCT |
| *H2-R* | GAATAGAGTAGATGCCGACCGCGGGTT |
| *BD-AaSlt2-F* | ATGGCCATGGAGGCCGAATTCatgggcgacctcgccaac |
| *BD-AaSlt2-R* | CCGCTGCAGGTCGACGGATCCTCATCGCATGCGACCGTCAA |
| *BD-AaSwi6-F* | ATGGCCATGGAGGCC GAATTC atgtacgccc aatctcccca |
| *BD-AaSwi6-R* | CCGCTGCAGGTCGAC GGATCC TTACTCTACG CCCTCAACCT |
| *AD-AaSwi6-F* | CCATGGAGGCCAGT GAATTC atgtacgccc aatctcccca |
| *AD-AaSwi6-R* | ACGATTCATCTGCAG CTCGAG TTACTCTACG CCCTCAACCT |
| *AD-AaMbp1-F* | CCATGGAGGCCAGT GAATTC ATGCCCCCCGCGCCCGAT |
| *AD-AaMbp1-R* | ACGATTCATCTGCAG CTCGAG CTATAGCGGACTTCCATCCAGTCC |
| *AD-AaRlmA-F* | CCATGGAGGCCAGT GAATTC atgggt cgcagaaaga tcga |
| *AD-AaRlmA-R* | ACGATTCATCTGCAG CTCGAG TCAGGTTTTG ACCCGCTTTT GG |

Primer sequences for constructing *AaSwi6* and *AaMbp1* knockout vectors, as well as primer sequences for yeast two-hybrid vectors of AaSwi6, AaMbp1, AaSlt2, and AaRlmA.

**Table S2 Standard information and standard curve**

| **Toxins** | **Compounds** | **Adducts** | **Time/min** | **Standard curve** | **R^2^** |
| --- | --- | --- | --- | --- | --- |
| AME | C_15_H_12_O_5_ | [M+H]^+^ | 4.115 | Y=2.371551X+13.451 023 | 0.9998 |
| AOH | C_14_H_10_O_5_ | [M+H]^+^ | 3.125 | Y=2.547454X+11.387 593 | 0.9994 |
| TeA | C_10_H_15_NO_3_ | [M+H]^+^ | 3.102 | Y=2.764007X+2.815 021 | 0.9999 |
| TEN | C_22_H_30_N_4_O_4_ | [M+H]^+^ | 3.053 | Y=65.262738X+30.167 858 | 0.9999 |

**Table S3 HPLC conditions**

| **Time/min** | **Fraction A/%** | **Fraction B/%** | **Flow/(mL.min^-1^)** |
| --- | --- | --- | --- |
| 0.00 | 95 | 5 | 0.3 |
| 1.00 | 45 | 55 | 0.3 |
| 4.00 | 15 | 85 | 0.3 |
| 5.00 | 95 | 5 | 0.3 |
| 7.00 | 95 | 5 | 0.3 |

The HPLC conditions are as follows: Agilent 6460 UHPLC system equipped with an Elipse Plus C_18_ column (1.8 μm, 2.1 mm × 150 mm); column temperature set at 30 °C; mobile phase A is 0.2% formic acid in water, and B is acetonitrile with 0.2% ammonia added; injection volume is 5 μL.

**Table S4 Primers used for gene expression analysis**

| Gene | Primer sequences (5' - 3') |
| --- | --- |
| *qRT-AaSwi6-F* | AATGGCATCCTTCAGTCAG |
| *qRT-AaSwi6-R* | TCAAGTCTTTGTTGCTCCTG |
| *qRT-AaMbp1-F* | CCTACAGTAATGTTCCCGTCTA |
| *qRT-AaMbp1-R* | AGGATGTGAGTGGCGTTG |
| *qRT-AaRlmA-F* | *AGCAAGCCAAGATTGAGGGTG* |
| *qRT-AaRlmA-R* | *GGCAGGACTACGCCAGAGGA* |
| *qRT-AaGPI1-F* | GGGGATGACGTGGAAGATGG |
| *qRT-AaGPI1-R* | TGATAGTCCGCAGCAACAGC |
| *qRT-AaGPI2-F* | CTCTGGAAACGCCGATGGGTC |
| *qRT-AaGPI2-R* | TGCGGAAGTCGCTGAGGTGA |
| *qRT-AaGPI3-F* | AACATTCAGTGGGAGACGACC |
| *qRT-AaGPI3-R* | CGTGCCTGATGCCCAAATAC |
| *qRT-Aachs1-F* | TCGCACTAATAACCATCTACGG |
| *qRT-Aachs1-R* | TCTTGCTCCTGCTCCACT |
| *qRT-Aachs2-F* | GCGGAGAAGGGCATTGTG |
| *qRT-Aachs2-R* | CGACGAGCGGGTTGAGAA |
| *qRT-Aachs3-F* | TCAGCCAGCGAGAAGCAA |
| *qRT-Aachs3-R* | GCAGAACCAAAGCCGACA |
| *qRT-Aachs4-F* | GCTCTGCGGCGAAACAAA |
| *qRT-Aachs4-R* | CTCGGAGTAATGCTCAACAACA |
| *qRT-Aachs6-F* | GCCAGGTAACCGAGGAAA |
| *qRT-Aachs6-R* | CAGGCAAACAGGTGACACTA |
| *qRT-Aachs7a-F* | TCGGTGATATTTCGTTTGG |
| *qRT-Aachs7a-R* | CTTGGTCGCAGATGGTGT |
| *qRT-Aachs7b-F* | CAATAGACAACAACGGATGG |
| *qRT-Aachs7b-R* | AGGAACAGGGTGGTGAGG |
| *qRT-Aachs8-F* | ACGGTAAGGATGGAAACG |
| *qRT-Aachs8-R* | GCACAGGGAACAGATTGG |
| *qRT-AaFKS-F* | CCGACGACTGGCTAAACT |
| *qRT-AaFKS-R* | ACAGCGGCGTGATGACTT |
| *qRT-AaGlu1-F* | CGAGGAGATTGTCATGGAGTG |
| *qRT-AaGlu1-R* | GATTTGCGGTGTTCGGTAG |
| *qRT-AaGlu2-F* | GAATGTCGATTAACCGTGAC |
| *qRT-AaGlu2-R* | CTGACCTCGTTTCCAGCA |
| *qRT-AaGlu3-F* | ACACCCTTCGCACTCGTA |
| *qRT-AaGlu3-R* | CCAGGTAGGCAATTTGGA |
| *qRT-AaKTR1-F* | CTGAGCCGCTGCGACGAGTT |
| *qRT-AaKTR1-R* | AAGGATTGTGGCGTTGATGC |
| *qRT-AaKTR2-F* | TCAAATTCTTCTGCGACATCA |
| *qRT-AaKTR2-R* | GGTCTCCCTCAGTTCCTTCAC |
| *qRT-AaKTR3-F* | GCACAAGCGGACCCTATTCT |
| *qRT-AaKTR3-R* | GTTCTGCCCTTCGTTGACCT |
| *qRT-Aabrm1-F* | ATGTTTGAGA AGAACGAATT |
| *qRT-Aabrm1-R* | CTACAACGCC TGACCAGT |
| *qRT-Aabrm2-F* | CTTGACCGTGGAAGGAAATCA |
| *qRT-Aabrm2-R* | TTGGGTGGAGGATCGAAGTG |
| *qRT-AaCmrA-F* | ACAGTTCTTCGTCGGCCAAA |
| *qRT-AaCmrA-R* | CAGCTGCGATGGAGCTCAT |
| *qRT-Aates-F* | CATCTAAGTAGCGAGCAGC |
| *qRT-Aates-R* | GGAAAGAGGCAGGACAAT |
| *qRT-Aatas-F* | CAGGACCACCGCTGACTA |
| *qRT-Aatas-R* | GCATTCCCATCTGTAACTAAAC |
| *qRT-AaOmtI-F* | AAGCTATCGGTCACGACT |
| *qRT-AaOmtI-R* | ATCACGGATTCAGCACTC |
| *qRT-AaaohR-F* | CTCTTCTGCCCTCCTTATCC |
| *qRT-AaaohR-R* | CTGACACCCTACTGTTTCCTC |
| *qRT-AamoxI-F* | GATAACCCACAAATAACCTACA |
| *qRT-AamoxI-R* | GTCCGTGACCAAAGACCAT |
| *qRT-AasdrI-F* | GAGATGGGACGAGGTTTC |
| *qRT-AasdrI-R* | CTATTGCCCTGATGGACT |
| *qRT-AalaeA-F* | TTGACATAGGGTGCGGAACT |
| *qRT-AalaeA-R* | CGGGCTGGATGGGACTTA |
| *qRT-AaVeA-F* | GCCCAAGTTCAACCCACA |
| *qRT-AaVeA-R* | CAACCATGCGTTCATTCAG |
| *qRT-AaVelB-F* | GGCGGGACAATACCAAAT |
| *qRT-AaVelB-R* | CCTGTAGCACGAACCAAAA |
| *qRT-AaVelC-F* | GGTGCCGCAGAGCAAGAA |
| *qRT-AaVelC-R* | GCCTGTCGGGCTGTATGA |
| *qRT-AaVosA-F* | ATTTCAAGCGACAAGTTCA |
| *qRT-AaVosA-R* | GTTGCTCATCATCCCTCT |
| *qRT-AapksA-F* | TGGTAACACCGCGGTCATC |
| *qRT-AapksA-R* | GCTCACTGTCACCATGTGCAT |
| *qRT-AapksC-F* | ACCGCTCCTTCCCAGTCT |
| *qRT-AapksC-R* | AGCACCTCGCTCGATACG |
| *qRT-AapksD-F* | GATGACGCACCACTGACT |
| *qRT-AapksD-R* | CGACAAGCACCGTAGAAC |
| *qRT-AapksE-F* | ATAATGAGGCGGTGTCGT |
| *qRT-AapksE-R* | CGGTGGTGCTTCAGTTTG |
| *qRT-AapksF-F* | GGTAGACTGGGACTTTGAGAC |
| *qRT-AapksF-R* | AGCGTGTTGAGAAGGTGG |
| *qRT-AapksG-F* | TGATGAGTGCGAGGAACG |
| *qRT-AapksG-R* | AACTGATGCCTCTGCGTAT |
| *qRT-AapksH-F* | CGAGAAAGCCAACCCGAATA |
| *qRT-AapksH-R* | GTGCCGCCGTAACCAAAA |
| *qRT-AapksI-F* | TGTGAAGGAGGAACAGCG |
| *qRT-AapksI-R* | GAAAGGCAAGCACCAGTA |
| *qRT-AapksJ-F* | TGTTCGTACTATCCGCATTT |
| *qRT-AapksJ-R* | GCTTCTCAGCCAAGGTGT |
| *qRT-AaCut1-F* | CTGATCTAGGCTCGAATGCTC |
| *qRT-AaCut1-R* | GCTATAACCGCTGTACCTTGACT |
| *qRT-AaCut2-F* | GTAAAGGGTGTCTTCCTCATCG |
| *qRT-AaCut2-R* | GAGTGCTGCGGAAAGTCCAT |
| *qRT-AaCut3-F* | GCCCTGTTGAGATCGCTGAG |
| *qRT-AaCut3-R* | AGGCGGACTTGACCGAGTTT |
| *qRT-AaCut4-F* | AGGCTATCCGCGAGAAGC |
| *qRT-AaCut4-R* | CCACCGAGGTTGGCATTA |
| *qRT-AaCL-F* | CAACTTGCCCGATCGTGATT |
| *qRT-AaCL-R* | TGTAGATCTCGAGGCCGTTGT |
| *qRT-AaβG1-F* | GTGGCACAACGGTGACAAAC |
| *qRT-AaβG1-R* | GGCAAGTGAGCGAACAGGAC |
| *qRT-AaβG2-F* | GCACTCCCGTCGCCATTACA |
| *qRT-AaβG2-R* | ATCGCCTTCAAGCCCTCATT |
| *qRT-AaβG3-F* | TTTTACGAGTTATGGTGGGC |
| *qRT-AaβG3-R* | CGCATAGAGCGATGAGAAGG |
| *GAPDH-F* | CTTACTGCCTCCACCAACTG |
| *GAPDH-R* | TGACGTTGGAAGGAGCGAAG |

Quantitative primer sequences related to cell wall synthesis, melanin synthesis, toxin synthesis, secondary metabolite synthesis, and cell wall-degrading enzyme genes in *A. alternata.*

**Fig. S1.** Identification of Δ*AaSwi6* and Δ*AaMbp1* strains of *A.alternata*. (A) Primers AaSwi6-F/R were used for PCR verification of the target gene fragments of the three transformants AaSwi6-15, AaSwi6-19, and AaSwi6-21. (B) Primers H1-F and H2-R were used for PCR verification of the HPH fragment in the three transformants AaSwi6-15, AaSwi6-19, and AaSwi6-21. (C) The Δ*AaSwi6* strains were confirmed by RT-qPCR, Δ*AaSwi6-15* and Δ*AaSwi6-21* were successfully constructed mutants. (D) Primers AaMbp1-F/R were used for PCR verification of the target gene fragments of the three transformants AaMbp1-13, AaMbp1-37, and AaMbp1-54. (E) Primers H1-F and H2-R were used for PCR verification of the HPH fragment in the three transformants AaMbp1-13, AaMbp1-37, and AaMbp1-54. (F) The Δ*AaMbp1* strains were confirmed by RT-qPCR, Δ*AaMbp1-37* and Δ*AaMbp1-54* were successfully constructed mutants.

**Fig. S2.** Infection related morphogenesis in the *A. alternata*. S: spore, G: germ tube, APP: appressorium-like.
